# Design, Modeling, and Validation of a Compact, Energy-Efficient Mixing Screw for Sustainable Polymer Processing

**DOI:** 10.3390/polym17020215

**Published:** 2025-01-16

**Authors:** David O. Kazmer, Stiven Kodra

**Affiliations:** 1 Department of Plastics Engineering, University of Massachusetts Lowell, Lowell, MA 01854, USA; 2 Department of Mechanical Engineering, University of Massachusetts Lowell, Lowell, MA 01854, USA; stiven_kodra@uml.edu

**Keywords:** plasticating screw extrusion, modeling, simulation, dispersive mixing, distributive mixing, thermal mixing, validation

## Abstract

This study presents the design, modeling, and validation of a mixing screw for energy-efficient single-screw extrusion. The screw features a short length-to-diameter (L/D) ratio of 8:1 and incorporates double flights with variable pitch and counter-rotating mixing slots. These features promote enhanced plastication by breaking up the solid bed and improving thermal homogeneity through backflow mechanisms relieving a 3.75 compression ratio. Non-isothermal, non-Newtonian simulations modeled the thermal and flow behavior for high-impact polystyrene (HIPS) and recycled polypropylene (rPP) under various operating conditions. Experimental validation was conducted using a 20 mm pilot-scale extruder with screw speeds ranging from 10 to 40 RPM and barrel temperatures of 220 °C and 240 °C. Results showed a strong linear dependence of mass output on screw speed, with maximum mass throughputs of 0.58 kg/h for HIPS and 0.74 kg/h for rPP at 40 RPM. Specific energy consumption (SEC) was calculated as 0.264 kWh/kg for HIPS and 0.344 kWh/kg for rPP, corresponding to efficiencies of 31.5% and 56.5% relative to theoretical minimum energy requirements. Compared to traditional general-purpose and barrier screws with L/D ratios of 27:1, the mixing screw demonstrated improved energy efficiency and reduced residence time distributions. These findings suggest the potential of the mixing screw for compact extrusion systems, including 3D printing and other sustainable polymer and bioplastics processing applications.

## 1. Introduction

Extrusion is a workhorse for the global plastics industry, converting polymer feedstocks into compounded materials as well as a wide array of products such as films, sheets, tubing, and profiles. Over the past decades, the pursuit to enhance throughput and mixing has driven a persistent trend toward higher length-to-diameter (L/D) ratios in plasticating screws with modern designs commonly extending to 30:1 or more. These longer screws are characterized by increased internal volume that facilitates greater heat transfer and shear-induced mixing while ensuring sufficient residence time at increased screw rotational speeds. However, these gains require larger and more expensive machines that also incur higher heat losses to the environment as well as broader residence time distributions. As such, this article investigates a mixing screw design that seeks energy efficiency and residence time uniformity relative to theoretical limits.

### 1.1. Overview of Related Work

The fundamental challenge in single-screw extrusion is the balancing of throughput, energy efficiency, and product consistency. Plastication screws are typically characterized by three primary functional zones along their length: the feed zone, the compression (or transition) zone, and the metering zone. In the feed zone, solid particulates are conveyed forward by the screw’s rotation, ensuring a consistent supply of material to subsequent sections. As the material progresses into the compression zone, it undergoes compaction and begins to melt, driven by a combination of external barrel heating and internal shear heating. This zone is critical for homogenizing the polymer melt and reducing voids or air pockets within the material. Finally, in the metering zone, the melt is further homogenized and pressurized, enabling a uniform and controlled flow through the die to achieve the desired product dimensions and properties.

The performance of the extrusion process depends on multiple factors, including screw geometry (e.g., length-to-diameter ratio, compression ratio, and pitch), barrel temperature profiles, screw rotational speed, and the rheological characteristics of the processed polymer. Early foundational work, such as that of Carley and McKelvey [1] as well as Tadmor and Klein [2], established the fundamental principles of these mechanisms, providing insights into heat transfer, material flow, and melting dynamics within the extruder. Melting within single-screw extruders is a complex process influenced by a combination of heat transfer, material flow, and mechanical shear. The classic melting model described by Tadmor, Duvdevani, and Klein [3] begins with the formation of a melt film at the barrel wall, where heat from the heated barrel surface is conducted into the solid polymer. This film is subsequently scraped off by the rotating screw flight, creating a pool of molten material adjacent to the solid bed. Shear forces and pressure within the channel further contribute to the melting process, with the material in the molten pool recirculating to transfer additional heat to the solid bed.

Subsequent research expanded upon these principles to account for alternative melting behaviors observed under specific processing conditions. For highly filled materials or those with poor thermal conductivity, conduction and localized melting within the polymer mass, rather than at the barrel wall, may dominate the process [4]. In starve-fed extrusion, where the screw is only partially filled, melting occurs through dispersed mechanisms with enhanced mixing and thermal homogeneity [5]. Experimental and computational studies have revealed the persistence of vortical flows and coiled sheet morphologies in the melt, phenomena that broaden residence time distributions and affect energy efficiency [6].

These findings highlight the complexity of melting mechanisms and underscore the importance of improved screw designs for material processing. General-purpose screws [7], with their uniform pitch and compression ratio, are versatile but often result in broad residence time distributions and inconsistent melting. To address these limitations, barrier screws, introduced by Maddock [8], incorporate a secondary flight to separate the molten polymer from the unmelted solid bed. This design prevents the insulating effects of the melt pool, ensuring more consistent plastication and improved mixing. Higher compression ratios promote melting [9] but may exacerbate shear-induced heating. Mixing elements like Maddock [10], rhomboidal [11], and confusor [12] sections increase distributive and dispersive mixing [13,14].

Plasticating screws have long been investigated and their designs improved through modeling and simulation. Early models, such as those by Carley and Strub [15] derived fundamental theories to optimize screw compression ratios and channel geometries. Modern tools such as computational fluid dynamics [16,17], finite element methods [18,19,20,21,22], and the discrete element method [9,23,24] have enabled high-fidelity modeling of complex phenomena including melt film formation, particle interactions, and shear-induced heating. These techniques assist the optimization of screw designs for specific materials and processes, significantly reducing the reliance on empirical trial-and-error methods.

### 1.2. Motivation for Current Work

Despite these advances in plasticating screw design and modeling, extrusion processes remain constrained by inefficiencies in melt homogenization, energy consumption, and residence time variability. These limitations are further exacerbated by the heterogeneity of feedstocks, particularly in recycled and filled polymers.

The mixing screw presented in this study is designed to address these limitations by incorporating a compact design with a high compression ratio in which most of the screw acts as a plasticating mixer using double flights of variable pitch with slots provided on a counter-rotating helix. The design minimizes residence time and distribution by breaking up the solidified bed and enhancing heat transfer. The screw’s short length-to-diameter ratio not only improves energy efficiency but also enables compact extruders with a lower carbon footprint that are suitable for use in 3D printing and other systems. The work thus aims to provide a new general-purpose design that bridges the gap between high-efficiency extrusion and sustainable material processing.

## 2. Materials and Methods

### 2.1. Modeling and Characterization of Screw Metering

**Figure 1:**
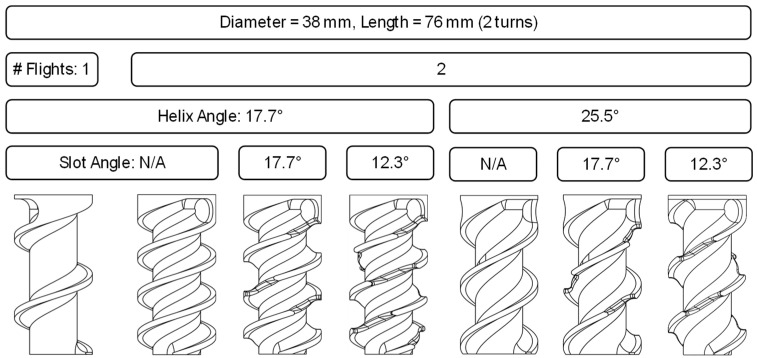
Design of prototype mixing screw metering sections showing variations in flight and slot geometries.

Representative screw sections were designed and fabricated to evaluate the influence of flight and slot geometries on screw metering. As shown in Figure 1, seven designs were investigated to characterize the effect of the number of flights, the flight helix angles, and the slot helix angles. Specifically, single and double-flighted screws were implemented with helix angles of 17.7° and 25.5°, respectively, while the mixing slots were configured with helix angles of either 12.3° or 17.7° or omitted altogether. For example, the leftmost design corresponds to a section of a general-purpose screw with a flight pitch equal to the screw diameter and a channel depth equal to 10% of the diameter. By comparison, the rightmost design provides a double-flighted screw with a 25.5° flight helix angle and mixing slots provided on a helix angle of 12.5°.

The objective was to quantify the impact of flight and slot designs on material throughput under controlled conditions. Flow simulations were conducted to investigate the predictive capability of these designs. The simulations were conducted with SolidWorks Flow 2023 using a finite volume method with a rotating outer wall to model the relative rotation of the screw relative to the barrel. The viscosity of low-density polyethylene (Dow 501i, Midand, TX, USA, melt flow rate 1.9) was modeled with a second-order polynomial equation. The mesh was created with a very fine (5 of 7 levels) using an option for advanced channel refinement providing a total of 432,198 cells (267,588 fluid elements and 164,610 solid elements).

**Figure 2:**
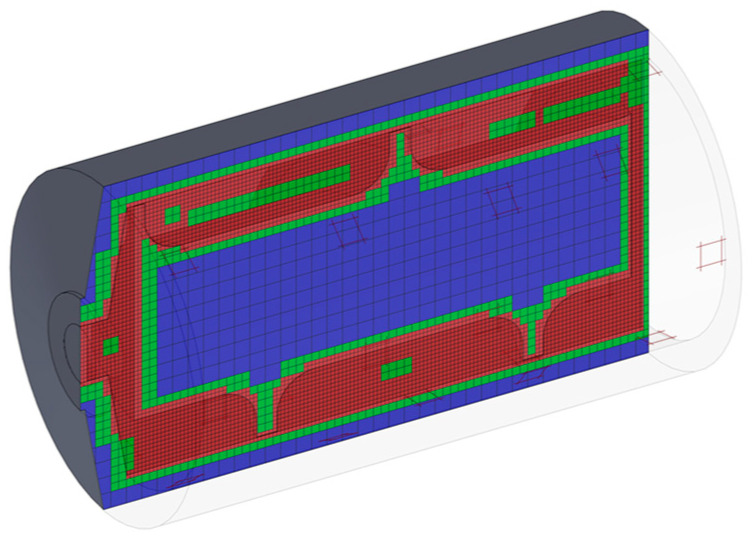
Cut isometric view showing cross-section of a typical mesh and rotating barrel used in simulation studies for the design shown at left in Figure 1.

A representative mesh is shown in Figure 2. The boundary conditions included a rotating barrel wall to simulate the screw rotation, atmospheric pressures at both the inlet and outlet, and a uniform temperature of 240 °C at the inlet and barrel walls. Material properties included a 3rd-order polynomial viscosity model [25] as well as a density of 750 kg/m^3^, specific heat of 2300 J/(kg·K), and thermal conductivity of 0.24 W/(m·K). A mesh refinement study was conducted with the results provided in Table 1; the results show that the mesh was adequate to minimize mesh size effects. Specifically, the transition from a ‘Fine’ to an ‘Extremely fine’ mesh level resulted in only a slight variation in flow rate (from 4217 mm^3^/s to 4150 mm^3^/s) and barrel torque (from 4.199 Nm to 4.217 Nm), indicating that further refinement beyond the ‘Very fine’ mesh does not significantly affect the simulation outcomes.

The barrel with an integrated hopper as well as all the designed mixing screw sections of Figure 1 were produced using selective laser sintering (SLS) of polyamide 12 (PA12) by Autotiv (Salem, NH, USA). The surface roughness of the SLS-produced parts typically ranges from 10 to 20 microns, which may influence the flow dynamics by increasing friction between the polymer melt and the screw surfaces, potentially affecting the material processing efficiency. The received parts had diametral tolerances on the order of 0.1% of the designed dimensions. Given a nominal diametral clearance of 0.2% between the screw and barrel bore, the assemblies exhibited a tight sliding fit that was operable with the extruder motor without leakage between the screw flight and barrel wall.

The performance of these mixing screw sections was assessed through a designed experiment using polypropylene (PP) pellets. Tests were conducted at screw speeds of 14 RPM and 34 RPM. Three samples were collected across one-minute intervals for each design and screw speed. Mass flow rates were measured by weighing the extruded material after each timed interval. Observed deviations between experimental and simulated results were analyzed, attributing discrepancies to the granularity of feedstock and geometry-induced flow variations. This integrated approach facilitated a comprehensive evaluation of the prototyped metering sections, providing insights into their performance and guiding future screw design.

### 2.2. Modeling and Characterization of Mixing Screw Behavior

A pilot-scale extruder was designed and tested with a screw diameter of 20 mm and an active length of 160 mm, corresponding to a length-to-diameter (L/D) ratio of 8:1. The design details for the double-flighted, variable-pitch screw are provided in Table 2. The design provides a rapid progression from the feed section to the plastication and metering stages. In the feed section (turn 1), the screw features a pitch of 36 mm, a flight width of 3 mm, and a channel depth of 4 mm, with a helix angle of 29.8°. This configuration maximizes volumetric capacity, ensuring efficient material intake.

As the material advances through the plastication sections (turns 2 through 6), the screw geometry transitions to increase compression and promote melting. The channel depth decreases from 3.6 mm at turn 2 to 2.0 mm at turn 7, while the channel width narrows from 15 mm to 8.8 mm. Concurrently, the pitch reduces from 36 mm to 22 mm. These changes in the screw channels increase the compression ratio [26] to a maximum of 375% in the metering sections. This relatively high compression ratio causes the screw to generate the shear and heat necessary to plasticate the material effectively, ensuring a smooth transition from solid to molten states.

**Figure 3:**
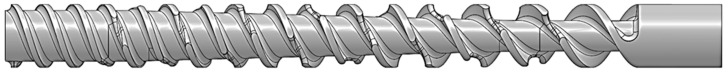
Shaded right view of the designed and validated mixing screw with specified variable-pitch and mixing-slot features.

The screw geometry incorporates a set of mixing slots arranged along a counter-rotating helix with a helix angle of 20°, starting at the second screw turn. These mixing slots are designed with a depth equal to 50% of the local channel depth and a width equal to 25% of the screw diameter. The slots divert approximately 25% of the material flow into the rearward channel, thereby enhancing mixing and promoting thermal homogeneity. The double-flighted configuration, used with the non-overlapping mixing slots, ensures complete wiping of the barrel wall to increase heat transfer while avoiding material stagnation. In the metering section (turns 7 and 8), the design adopts a pitch of 20 mm, a flight width of 2 mm, and a channel depth of 2 mm to deliver flow at pressure. The resulting screw design is illustrated in Figure 3. Both the screw and the barrel were fabricated from grade 316 stainless steel to ensure reliability in validation. The 3D CAD design is provided in the Supplementary Materials along with a video of extruder operation, results and related Matlab analyses. 

The extruder incorporated a 24 V, 172 W brushless DC (BLDC) motor providing 17.6 Nm of output torque when coupled to a 50:1 high precision gearbox (Stepper Online 57BLR70-24-02-HG50, Nanjing City, China). The motor was controlled by a motor driver (Stepper Online BLD-510B) rated to yield a maximum speed of 70 RPM, but in operation, the maximum operable screw speed was found to be 45 RPM. Three heater bands totaling 700 W were spaced uniformly along the barrel (average power density of 30 W/cm^2^) and were controlled via a solid state relay given feedback from a single type K thermocouple located at the metering zone. Both the motor driver and solid state relay were controlled with pulse width modulation at 200 Hz via an Arduino R4 Wifi microcontroller.

**Figure 4:**
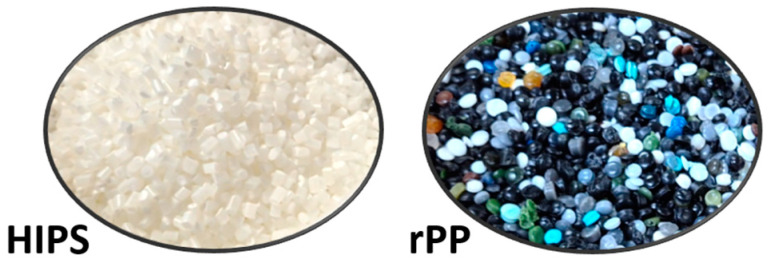
Photographs depicting 50 mm wide section of HIPS (**left**) and recycled polypropylene (rPP, **right**).

Experimental validation included mass output and melt temperature measurements for high-impact polystyrene (HIPS [27]) and recycled polypropylene (rPP [28]) feedstocks shown in Figure 4; both these materials are from the same lots of materials as the cited publications also related to extrusion. The high-impact polystyrene (HIPS, left) is smaller and more uniform in size while the recycled polypropylene (rPP) displays significant variation in size, color, and composition, reflecting the heterogeneous nature of recycled material streams. Each material was tested at four screw speeds (10, 20, 30, and 40 RPM) and two barrel temperatures (220 °C and 240 °C). After 15 min of extrusion for each material and temperature combination, the melt temperature was probed at each screw speed with an immersive thermocouple placed directly into the melt stream during extrusion, while mass output was measured based on the extruded material collected over one minute with five replicates.

The energy efficiency in the article is based on the specific energy consumption (SEC) measured during the extrusion process. The SEC is defined as the total energy, E, consumed per unit mass, m, of polymer processed, which includes both the mechanical energy required to drive the extruder and the thermal energy used to heat the polymer to its processing temperature. The minimum SEC is estimated from the mass flow, specific heat capacity $C_{P}$, melting enthalpy h (for semicrystalline polymers), density ρ, imposed temperature change ∆T, and imposed pressure change ∆P required to extrude the melt according to the equation:

(1) $$SEC=\frac{E}{m}=C_{P}∆T+h+\frac{m}{\rho }∆T$$

The energy efficiency is then calculated by dividing this minimum SEC by the actual energy consumed during the process as measured with an energy meter (Poniie PN2000).

**Figure 5:**
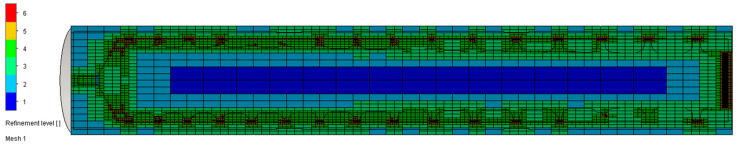
Cut isometric view showing cross-section of mesh and rotating barrel.

Computational modeling was also conducted using the finite volume method to simulate the behavior of high-impact polystyrene (HIPS, BASF 486F) under extrusion conditions. Boundary conditions for the simulations were configured with an inlet temperature of 140 °C and a metering zone barrel temperature of 240 °C, with atmospheric pressure set at both the inlet and outlet. The barrel wall was rotated to simulate screw movement. As shown in Figure 5, the refined mesh incorporated over 1,038,201 total cells (533,822 fluid and 504,379 solid) to ensure reasonable accuracy. Simulations were conducted with SolidWorks/Flow at screw speeds ranging from 15 to 180 RPM.

## 3. Results

### 3.1. Metering Behavior of Prototyped Mixing Screw Sections

The metering study investigated the mass output of prototyped mixing screw sections as a function of screw geometry and operating conditions. Experimental observations were compared with computational simulations to identify the primary factors influencing mass output and to evaluate the predictive capability of the simulation model. This dual approach provided insights into the effectiveness of various design configurations and operating conditions in optimizing screw performance.

Multiple regression modeling was conducted using the *lm*() function in Matlab, which fit linear models to the data and provided estimates of coefficients and their statistical significance. The model included screw speed (RPM), number of flights (1 or 2), flight helix angle (17.7° or 25.5°), and the number of mixing slots (ranging from 0 to 8, determined by the flight and mixing slot helix angles defined in Figure 1). The analysis identified two significant two-way interactions: (1) Helix Angle * Number of slots, and (2) RPM * Number of slots. The intercept and all other interaction terms were excluded from the model. The resulting model coefficients and their associated *p*-values are summarized in Table 3.

Both multiple regression models have a high predictive capability, with *R*^2^ of 0.9956 for the simulation and 0.9306 for the experimentation with pellets. The root mean square error (RMSE) indicates the difference between the modeled main effects and the results for each screw section (including sample-to-sample variation for the experimentation). All model *p*-values are well below the 0.05 value corresponding to a 95% confidence level. Given the consistent model form and adherence of the simulation to experimental conditions, the coefficients between the simulation and experimental may be directly compared.

**Figure 6:**
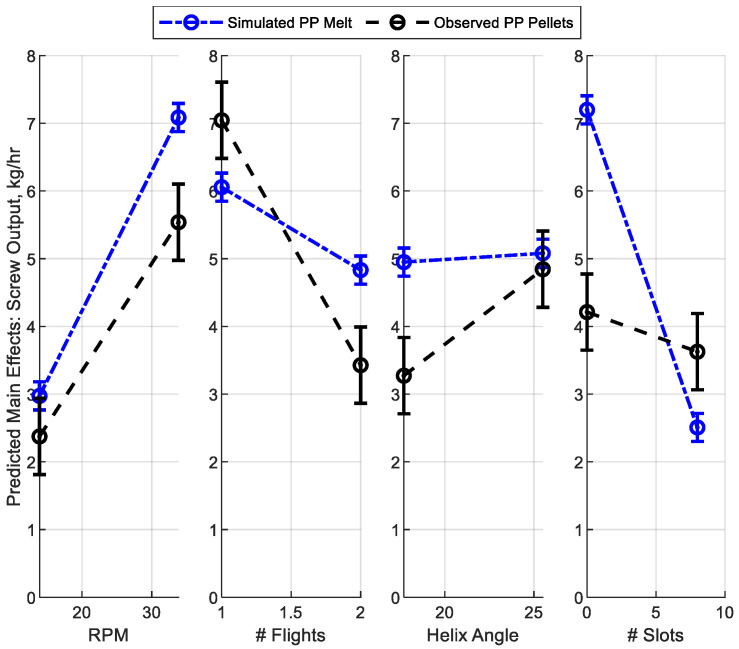
Main effects on mass output for melt simulation (blue dotted) and pellet experimentation (black dashed) as functions of screw speed, number of flights, helix angle, and mixing slots, with error bars representing the RMSE.

Figure 6 graphs the modeled main effects for mass output (kg/h) as a function of the screw speed (RPM) and mixing screw design parameters including number of flights, helix angle, and number of mixing slots. The results are shown for simulated melt data (dotted blue line) and observed pellet data (dashed black line), allowing direct comparison of predictions with experimental observations. The traces of Figure 6 also include the effects of the interaction terms of Table 3 while the error bars represent the RMSE of the two models.

Overall, there is reasonable agreement between the simulation and experimental models, indicating that while the simulation is imperfect, it serves as a useful predictor of reality. Both the simulation and experimentation models suggest a strong linear increase in mass output with screw RPM, as would be expected given the material displacement along the screw with each turn. The experimentation with pellets was found to provide 30% lower mass output per screw turn than the simulation. This discrepancy is attributed to the lower packing density of the ellipsoidal pellets in the experiments, which contrasts with the uniform, fully dense polymer melt assumed in the simulation.

The models showed more significant discrepancies in their predictions regarding the influence of mixing screw design parameters. Most notably, the simulation substantially underpredicted the reduction in mass output observed when transitioning from a single-flighted to a double-flighted screw. In practice, a 50% reduction in output was recorded, whereas the simulation predicted only a 17% reduction. Similarly, significant differences were observed with respect to the effect of the flight helix angle. While the simulation suggested a negligible impact, experimental results showed a 45% increase in flow rate when the helix angle was increased from 17.7° to 25.5°.

Significant differences between the simulation and experimental results were also observed regarding the effect of the mixing slots. The simulation predicted a 65% reduction in mass output for a double-flighted design with a slot helix angle of 12.3° (resulting in eight slots), whereas the experiments yielded only a 17% reduction. This discrepancy highlights the challenges of accurately modeling slot-induced flow disruptions in pellets, melts, and mixtures thereof. Two factors contributed to this disparity. First, the SLS-fabricated prototypes had a rough surface finish, with an *R*_*a*_ value of around 20 microns, which would inhibit the sliding of pellets along the screw surface. Second, the relatively large size of the pellets, at around 40% of the slot height, would reduce the proportion of particles capable of backflow through the slots. Both factors likely diminished the extent of backflow, thereby reducing the observed effect of the slots on mass output.

### 3.2. Behavior of Plastication in Mixing Screw

**Figure 7:**
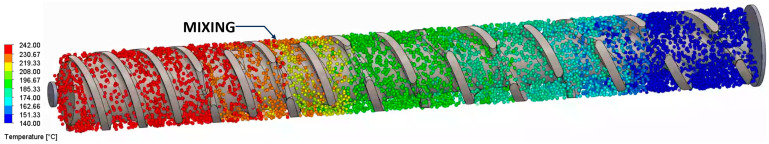
Particle distributions wherein color indicates material temperature and shows backflow.

The flow simulation results provide a detailed depiction of the plastication behavior facilitated by the implemented mixing screw design, emphasizing the interplay between thermal energy transfer and material flow dynamics. The particle distribution shown in Figure 7 captures the progression of processed material along the screw, with color coding representing material temperature and highlighting the presence of backflow phenomena. The image distinctly shows the material transitioning from cold near the inlet at the right (blue regions, ~140 °C) to hot near the outlet at the left (red regions, ~240 °C). This temperature gradient reflects the gradual plastication process, driven by thermal energy transfer through conduction and viscous dissipation as the material is conveyed. Backflow is particularly evident near the mixing slots, with particles recirculating into preceding screw channels. This recirculation enhances mixing and promotes thermal homogenization, as demonstrated by the diverse temperature gradients within the flow field (e.g., red, yellow, and orange near the end of the transition zone as indicated by the arrow).

**Figure 8:**
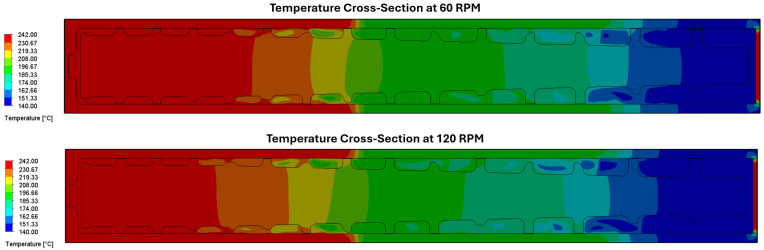
Cross-sections showing temperature distribution in barrel, melt, and screw at (**top**) 60 RPM and (**bottom**) 120 RPM.

The cross-sectional temperature distributions shown in Figure 8 illustrate the temperature distribution in the barrel, melt, and screw at operating speeds of 60 RPM (top) and 120 RPM (bottom). At 60 RPM, the temperature profile demonstrates a gradual progression from lower temperatures (blue regions, 140 °C) near the inlet to higher temperatures (red regions, 240 °C) near the outlet. Areas adjacent to the active screw flanks and mixing slots tend to exhibit higher temperatures, correlating with increased shear rates and higher heat conduction. The temperature distribution is very similar at 120 RPM, though the temperature contours shift approximately half a diameter to the left, meaning that the process material does not reach the target melt temperature of 240 °C until very close to the metering section. The reason is that at higher speeds, the more rapid conveyance of the cooler melt from the inlet begins to dominate the heat conduction and viscous dissipation, thereby determining an upper limit for the operating speed at which melt temperature variation can become an issue.

**Figure 9:**
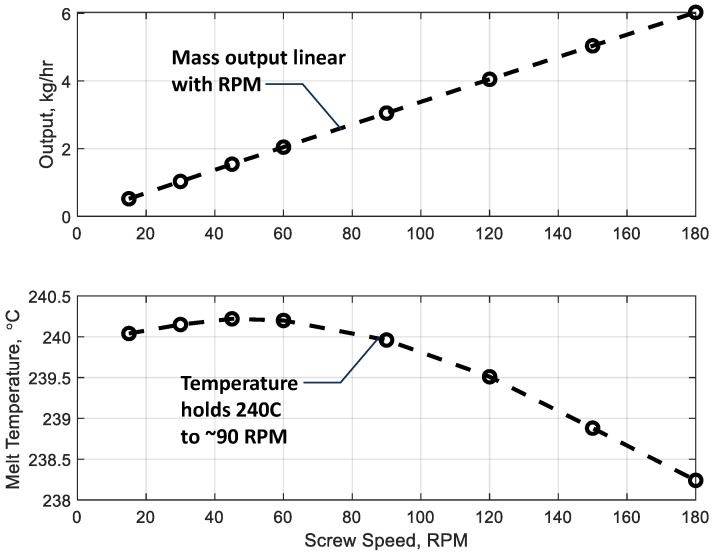
Predicted mass output (**top**) and bulk melt temperature (**bottom**) from the simulation as a function of screw speed.

Figure 9 illustrates the predicted relationship between screw speed and two key processing parameters: mass output (top) and bulk melt temperature (bottom). The mass output shows a strong linear correlation with screw speed, indicating that the screw design efficiently conveys material without significant losses or backflow across the studied range. The bulk melt temperature initially increases slightly above the set temperature of 240 °C at intermediate screw speeds due to viscous dissipation, which is proportional to the product of the viscosity and the square of the shear rate, providing significant internal heating. However, beyond 90 RPM, the bulk melt temperature begins to decline, suggesting that the rapid conveyance of cooler material from the inlet reduces the residence time available for heat transfer. Consistent with the advancing temperature contours of Figure 8, this decreasing temperature above 90 RPM indicates the existence of a critical operating limit where higher screw speeds could lead to insufficient thermal homogenization and extrudate inconsistencies.

**Figure 10:**
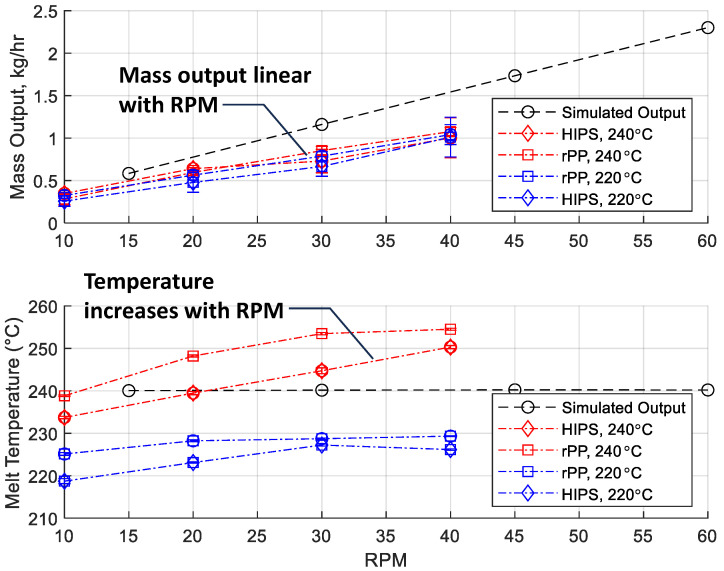
Observed mass output (**top**) and bulk melt temperature (**bottom**) from the experiments with HIPS (diamonds) and recycled polypropylene (rPP, squares) at 220 °C (blue) and 240 °C (red).

Figure 10 presents the experimental results for observed mass output (top) and bulk melt temperature (bottom) for high-impact polystyrene (HIPS, diamonds) and recycled polypropylene (rPP, squares) at set temperatures of 220 °C (blue) and 240 °C (red). The mass output exhibits a nearly linear dependence on screw speed, confirming the strong influence of RPM on throughput. At any given screw speed, the rPP consistently achieves higher mass output than HIPS, likely due to rPP’s lower melt viscosity, which facilitates easier conveyance through the screw channels. Additionally, operating at the higher set temperature of 240 °C yields slightly higher mass outputs across both materials, reflecting the lower viscosities and reduced flow resistance of both materials at higher temperatures.

Direct temperature measurement with an insertion probe into the melt stream showed that, for both HIPS and rPP, the melt temperature increases well above the set temperature due to the dominance of viscous dissipation. The magnitude of the temperature increase, around 10 °C, is much more significant than the peak 2 °C predicted by simulation. As the screw speed increases from 30 to 40 RPM, the melt temperature tends to plateau, indicating the potential onset of melt temperature decreases associated with increased melt convection requirements associated with higher rates of material conveyance and decreased residence times. Unfortunately, further investigation of this behavior at higher screw speeds was prevented given the use of a gearbox with a 50:1 transmission to ensure adequate shaft torque that limited the screw speeds to 45 RPM. Regardless, no instabilities in the extrudate output or melt temperature were observed suggesting that the process would be unstable at higher screw speeds.

The results’ statistical model coefficients and *p*-values are provided in Table 4. The model coefficients for the mass output indicate that the screw speed is the dominating factor, with the mass output increasing 23.8 g per hour per RPM, significant at a 99.9+% confidence level (*p* = 3.87 × 10^−11^). The coefficients for the intercept (−0.5274 kg/h, negative given the positive melt temperature coefficient and minimum melt temperature value of 220 C), material type (+0.04874 kg/h when switching from HIPS to rPP), and melt temperature (+0.002494 kg/h per °C) are significant at the 90% confidence level but not at 95%, with *p*-values of 0.0801, 0.0634, and 0.0584, respectively. The positive coefficient for material indicates that rPP yields slightly higher mass output compared to HIPS, likely due to rPP’s lower viscosity that facilitates better flow. Similarly, the positive coefficient for melt temperature suggests improved flowability and throughput at higher operating temperatures. The model explains 97.7% of the variation in the data with a root mean squared error (RMSE) of 0.0477 kg/h, indicating a high degree of predictive accuracy.

**Figure 11:**
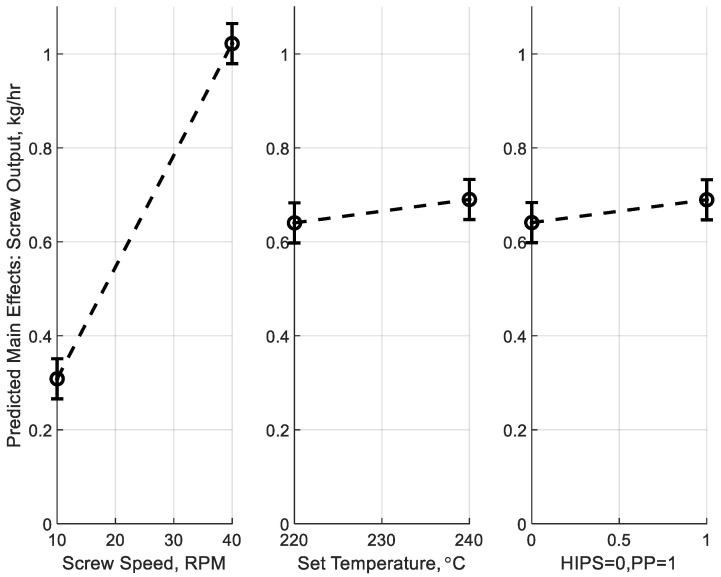
Main effects on mass output for melt extrusion with HIPS and rPP at varying screw speeds and set temperatures. Error bars represent the root mean square errors (RMSE) of the model including scatter across five replicates for each material and temperature combination.

Figure 11 illustrates the main effects of screw speed, melt temperature, and material type on mass output during melt extrusion of HIPS and rPP. The results confirm that screw speed is the dominant factor, as indicated by a steep linear increase in mass output with increasing RPM. Melt temperature also has a positive though lesser effect, with higher set temperatures resulting in a small increase in output due to reduced material viscosity. The material type similarly shows slightly higher output for rPP over HIPS, reflecting its lower resistance to flow. Error bars, representing the root mean square errors (RMSE) of the model and variability across replicates, are small relative to the main effects and thus verify the reliability of the model and consistency of the experimental results.

Energy consumption was monitored and analyzed for each combination of material and temperature settings, with specific energy consumption (SEC) calculated as the total energy consumed divided by the total amount of material processed. The regression model coefficients and their *p*-values are provided in the right-most column of Table 4; the model has good predictive accuracy, explaining 95% of the observed variance in SEC with a root mean square error (RMSE) of 0.0169. The model shows that screw speed most significantly impacts SEC with −0.003987 kWh/kg per RPM (*p* = 8.06 × 10^−5^), indicating a decrease in energy consumption per unit mass at higher speeds due to the more efficient throughput at elevated RPMs. The material type also has a significant effect on SEC, with rPP requiring 0.08164 kWh/kg more energy than HIPS (*p* = 4.44 × 10^−4^), consistent with rPP’s higher specific heat and melting enthalpy associated with its semi-crystalline solid state. The melt temperature effect has a minor effect (−0.001074 kWh/kg, *p* = 0.116) that is not statistically significant at the 95% confidence level. This result suggests that higher set temperatures may improve energy efficiency by assisting feeding and reducing viscosity, although the effect is minimal compared to screw speed and material type.

The energy efficiency of the extruder with the mixing screw was compared to prior works characterizing energy efficiency including a study [1] with a general purpose and barrier screw having a 27:1 length-to-diameter ratio in a 38 mm Davis-Standard extruder as well as a second study [3] comparing a molding machine with a 25 mm general purpose screw to 3D printing with filament-based material extruders. The results are provided in Table 5.

The extruder with the mixing screw in this work exhibited specific energy consumption (SEC) values of 0.264 kWh/kg for HIPS and 0.344 kWh/kg for rPP, representing the average across screw speeds of 10, 20, 30, and 40 RPM. These results correspond to efficiencies of 31.5% and 56.5%, respectively, relative to the minimum theoretical energy computed as the integral of the varying specific heat (including melting enthalpy) from room temperature to the set melting temperature. The mixing screw outperformed the 27:1 L:D general-purpose screw, which had efficiencies of 27.1% for HIPS and 47.6% for LDPE, averaged at higher screw speeds of 20, 40, and 60 RPM. Further, the general-purpose screw demonstrated poor melt consistency at 60 RPM with semi-molten pellets appearing in the melt stream. The barrier screw performed significantly better, ensuring good melt consistency at all screw speeds but still exhibited lower efficiencies of 20.7% for HIPS and 42.7% for LDPE compared to the mixing screw.

There are two likely reasons for the lower efficiency of traditional screws. The first and more obvious reason is that longer screws require longer barrels, which have greater surface area and consequently more heat transfer to the environment. The second and more subtle reason is that both general-purpose and barrier screws tend to develop a melt pool conveyed alongside a colder solidified bed. This phenomenon, described by Tadmor’s five-zone melting model, is inherently less energy efficient because the melt pool and the recirculating melt around the solidified bed tend to overheat, leading to vortical fountain flows [6], wide residence time distributions, and reduced energy efficiency. Furthermore, in the prior work, monitoring of the cooling fans on the extruder revealed that they were highly active in the barrier zone of the barrier screw when operated at 40 and 60 RPM. While the barrier screw demonstrates robust performance in achieving consistent melting, its excess shearing of the melt and increased heat dissipation to the environment contribute to its reduced energy efficiency.

## 4. Discussion

### 4.1. Further Screw Design Improvements

The differentiating design features of the described mixing screw include its very short length-to-diameter ratio and high compression ratio that are only operable with the addition of the mixing slots that allow backflow of the material. The plasticating behavior of the screw was highly effective, producing a homogeneous melt even when processing coarse recycled polypropylene pellets of varying sizes, colors, and compositions [28]. The feeding and metering functions of the screw were limited, however. Feeding was poor when the temperature of the barrel at the feed section was near room temperature but performed well at higher temperatures that supported higher friction between the barrel and process material. Melt pressures were adequate for the 2 mm diameter cylindrical orifice but could limit the mass output at smaller orifice diameters. Adding a turn in both the feeding and metering zones could resolve such issues. Alternatively, the mixing screw could be used with a metering pump to provide positive displacement control.

The mixing slot designs proved effective but are not claimed to be optimal. As a first attempt, the design strategy was to “shoot for the middle.” As such, the slot depth was literally set to half the local channel depth, while the slot width was set to half the screw radius. Further research is needed to optimize the size and number of the mixing slots. The hypothesis “if some is good, then more is better” implies that it may be possible to use even more mixing action and an even higher compression ratio to effectively plasticate materials with shorter screws while maintaining output with higher length-to-diameter ratios.

Further screw design optimization can be better supported with further plastication simulation. The most significant source of error is the improper material modeling in the feed and transition sections. In the feed section, the material consists of a mixture of air and feedstock leading to a lower density and mass flow rate compared to the simulations. Conversely, the material in the feed section can exhibit slip whereby the material rotates within the screw without significant axial motion, such that the simulation can overpredict the mass flow rate. Material modeling in the transition section is likewise more complex than simulated as the real material consists of islands of lower-temperature solids contained within a higher-temperature matrix of melt. High-fidelity modeling of these phenomena [9,23,24] can assist optimization of screw designs for specific materials and processes, significantly reducing the reliance on empirical trial-and-error methods

### 4.2. Screw Channel Width and Pitch Design Guidelines

The mixing screw is based on a new screw design guideline derived from the results of the metering study. First, characterizing the mass output as a function of a number of flights and helix angle led to the realization that the channel width should be a multiple, e.g., three or four times the channel depth to ensure effective shear stress transmission from the barrel to the processed material. This guideline, irrespective of the number of flights, implies that plasticating screws should avoid uniform flight pitches as in general-purpose designs. Instead, the flight pitch should vary along the screw’s length in proportion to the channel depth to optimize material processing. Similarly, the thickness of the flights can be reduced proportionally with the channel depth, maintaining structural integrity while maximizing channel volume and throughput efficiency. These strategies enabled the incorporation of eight full rotations of the flight helix in a double-flighted mixing screw that started out with an initial pitch equal to twice the screw diameter.

### 4.3. Applications

Table 4 also provided efficiency values for 3D printing and injection molding. Compared to screw-based extrusion, the filament-based 3D printing process had the poorest efficiency at 3.8%, largely limited by its low mass throughput and additional energy required for heating the print bed and driving the motors. The mixing screw was designed for immediate application as a pellet-based extruder for 3D printing and development in this area is ongoing. Injection molding is also a relatively low-efficiency process compared to extrusion. The reason is that the reciprocating screws in injection molding require extended barrel lengths that lose heat to the environment while also only operating with the material on an intermittent basis. It may be that extrude-to-fill type processes may be feasible wherein the extruder retains sufficient plasticated melt volume in a longer and deeper metering zone and then injects the material at moderate screw speeds, again assisted with a metering pump. This concept suggests the potential for combining continuous extrusion with intermittent injection operations, potentially bridging the efficiency gap between extrusion and injection molding processes.

### 4.4. Need for Enhanced Barrel Temperature Control

Although beyond the scope of this study, experimental observations highlighted the need for improved barrel temperature control. To minimize mass for integration with 3D printing, the mixing screw was paired with a compact barrel having a wall thickness of only 3 mm. When the screw speed was increased by 10 RPM, a 5 °C drop in barrel temperature was quickly detected by the barrel thermocouple due to the heat drawn by the higher conveyance of cooler material. This temperature excursion was resolved within a few minutes through the integral action of the PID controller. However, heating demand could be modeled as a function of screw speed to predict and preemptively adjust the supplied heating power, thereby avoiding such transient errors in dynamic applications like 3D printing or injection molding.

## 5. Conclusions

This study introduces an innovative mixing screw design optimized for single-screw extrusion, addressing critical challenges of energy efficiency and melt homogeneity. Featuring a short length-to-diameter ratio of 8:1, double flights with variable pitch, and counter-rotating mixing slots, this design significantly enhances backflow and compression to achieve notable improvements in mass outputs and energy efficiencies of 31.5% for HIPS and 56.5% for rPP, surpassing traditional general purpose and barrier screw designs. These outcomes highlight the screw’s capability to maintain consistent performance with complex, recycled polymers, underscoring its suitability for energy-conscious, sustainable polymer processing. The compact design also facilitates integration into constrained spaces like 3D printing setups. Future efforts will focus on (1) refining slot geometries for better mixing and applying the design across a broader spectrum of polymers to establish its broader applicability and impact on modern manufacturing, (2) integrating predictive heating models to improve barrel temperature control under dynamic operating conditions, and (3) applying the mixing screw design to a broader range of polymers including highly filled and bio-based materials towards modern sustainable polymer processing.

## Figures and Tables

**Table 1:** Mesh refinement study details including number of mesh elements and results.

Mesh Level	Number of Fluid Cells	Number of Solid Cells	Flow Rate, mm^3^/s	Barrel Torque, Nm
4 (Fine)	110,411	72,936	4217	4.199
5 (Very fine)	267,588	164,610	4189	4.215
6 (Extremely fine)	640,310	314,484	4150	4.217

**Table 2:** Design details for double-flighted, variable pitch screw prior to addition of mixing slots.

Screw Turn: Purpose	Flight Pitch, mm	Flight Width, mm	Channel Width, mm	Channel Depth, mm	Compression Ratio (%Feed)
1: Feed	36	3.0	15	4.0	100
2: Plastication	36	3.0	15	3.6	111
3: Plastication	28	2.8	11.2	3.2	167
4: Plastication	26	2.6	10.4	2.8	206
5: Plastication	24	2.4	9.6	2.4	260
6: Plastication	22	2.2	8.8	2.2	310
7: Metering	20	2.0	8	2.0	375
8: Metering	20	2.0	8	2.0	375

**Table 3:** Multiple regression models for mass output (kg/h) for simulation of melt and observation of pellets for significant factors and first-order interactions.

Model Term	Melt Simulation R^2^ = 0.9956; RMSE = 0.214	Pellet Experiments R^2^ = 0.9306; RMSE = 0.601
RPM	0.3080 *p* = 3.29 × 10^−83^	0.2138 *p* = 9.50 × 10^−19^
Number of flights	−1.224 *p* = 1.13 × 10^−23^	−3.616 *p* = 7.53 × 10^−13^
Helix Angle	0.1027 *p* = 1.87 × 10^−21^	0.2788 *p* = 7.82 × 10^−11^
Number of slots	0.5412 *p* = 4.19 × 10^−24^	0.7095 *p* = 2.30 × 10^−5^
Helix Angle × Number of slots	−0.02298 *p* = 1.71 × 10^−22^	−0.0206 *p* = 3.72 × 10^−3^
RPM × Number of slots	−0.02715 *p* = 1.74 × 10^−54^	−0.01470 *p* = 2.77 × 10^−6^

**Table 4:** Multiple regression models for mass output (kg/h) and specific energy consumption (kWh/kg) for plastication of HIPS and rPP pellets with described mixing extruder.

Model Term: Range [Units]	Mass Output [kg/h] R^2^ = 0.977; RMSE = 0.0477	SEC [kWh/kg] R^2^ = 0.950; RMSE = 0.0169
Intercept: [per column]	−0.5274 *p* = 0.0801	0.6622 *p* = 0.00160
Screw Speed: 10, 20, 30, 40 [RPM]	0.02378 *p* = 3.87 × 10^−11^	−0.003987 *p* = 8.06 × 10^−5^
Material: 0 = HIPS, 1 = rPP	0.04874 *p* = 0.0634	0.08164 *p* = 4.44 × 10^−4^
Melt Temperature: 220,240 [°C]	0.002494 *p* = 0.0584	−0.001074 *p* = 0.116

**Table 5:** Comparison of specific energy consumption for varied screw designs and applications including single screw extrusion (SSE), 3D printing (3DP), and injection molding (IM).

Process: Material [Reference]	Specific Energy Consumption kWh/kg (% Efficiency)	Comments
SSE: HIPS [This work]	0.264 (31.5%)	Screw speeds 10 to 40 RPM; would be better at higher RPM
SSE: HIPS [27]	0.307 (27.1%)	27:1 L:D general purpose screw at speeds of 20, 40, 60 RPM; low quality
SSE: HIPS [27]	0.403 (20.7%)	27:1 L:D barrier screw at speeds of 20, 40, 60 RPM; low quality
SSE: rPP [This work]	0.344 (56.5%)	Screw speeds 10 to 40 RPM; would be better at higher RPM
SSE: LDPE [27]	0.408 (47.6%)	27:1 L:D general purpose screw at speeds of 20, 40, 60 RPM; low quality
SSE: LDPE [27]	0.455 (42.7%)	27:1 L:D barrier screw at speeds of 20, 40, 60 RPM; low quality
3DP: PLA [29]	5.28 (3.8%)	Filament-driven 3D printing efficiency limited by throughput
IM: PLA [29]	0.929 (13.6%)	Molding more energy-intensive than extrusion

## Data Availability

The original contributions presented in this study are included in the article and Supplementary Material. Further inquiries can be directed to the corresponding author.

## Supplementary Materials

The following supporting information can be downloaded at: https://www.mdpi.com/article/10.3390/polym17020215/s1, simulation data “Simulation Results.csv”, video of extruder operation “20241019_ 094810_Line_HIPS_Puller_Extruder_Puller.mp4”, extrusion data “20241019 Completed Extruder Calibration and DOE.csv”, 3D CAD of mixing screw “20241216 Mixing Screw Design.STEP”, and Matlab analyses “Plot_Simulation_Results.m”, “PlotFit_MixoMaster.m”, and “PlotFit_MixoMaster_3Factors.m”.
